# Sleep Bruxism and Obstructive Sleep Apnea Are Not Risk Factors for Tension-Type Headache (TTH): A Polysomnographic Study

**DOI:** 10.3390/jcm13133835

**Published:** 2024-06-29

**Authors:** Bartłomiej Błaszczyk, Helena Martynowicz, Piotr Niemiec, Jakub Przegrałek, Martyna Staszkiewicz, Anna Wojakowska, Sławomir Budrewicz, Marta Waliszewska-Prosół

**Affiliations:** 1 Student Research Club No K133, Faculty of Medicine, Wroclaw Medical University, 50-556 Wroclaw, Poland; bartlomiej.blaszczyk@student.umw.edu.pl (B.B.); piotr.niemiec@student.umw.edu.pl (P.N.); jakub.przegralek@student.umw.edu.pl (J.P.); martyna.staszkiewicz@student.umw.edu.pl (M.S.); 2 Department and Clinic of Internal Medicine, Occupational Diseases, Hypertension and Clinical Oncology, Wroclaw Medical University, 50-556 Wroclaw, Poland; helena.martynowicz@umw.edu.pl (H.M.);; 3 Department of Neurology, Wroclaw Medical University, 50-556 Wroclaw, Poland; slawomir.budrewicz@umw.edu.pl

**Keywords:** TTH, bruxism, sleep apnea, OSA, sleep disturbance, polysomnography, symptoms, risk

## Abstract

**Background:** Tension-type headache (TTH) is the most common primary headache. Obstructive sleep apnea (OSA) and sleep bruxism (SB) are two of the most common sleep disorders; however, the relationship between TTH, OSA, and SB has not been conclusively proved in the literature. The objective of our study was to estimate potential associations with OSA and SB in TTH subjects. **Methods**: 108 adult individuals who underwent polysomnography (vPSG) were included, and the group was divided into two subgroups: TTH (*n* = 34) and control (*n* = 74). The International Classification of Headache Disorders (ICHD-3) guidelines were used to diagnose TTH. OSA and SB diagnoses were based on vPSG examination with electromyographic (EMG) recordings and the American Academy of Sleep Medicine (AASM) criteria. The results were analyzed, where *p* < 0.05 was considered to be statistically significant. **Results**: In the TTH group, the incidence of SB was more than two times lower than the control (OR = 0.41, 95% CI: 0.17–0.96, *p* < 0.05). However, the incidence of severe SB (BEI > 4) was similar in the TTH and control groups (OR = 0.54, 95% CI: 0.21–1.35, *p* > 0.05). Additionally, phasic and tonic SB episodes were less frequent in the TTH group compared to the controls (*p* < 0.05). The mean apnea–hypopnea index (AHI) was not significantly different between the TTH and control groups (*p* > 0.05). The sleep architecture and respiratory disturbances did not differ between the examined groups (*p* > 0.05). **Conclusions**: SB is not a risk factor for TTH. Moreover, severe SB is not connected with TTH. OSA is not a risk factor for TTH. Sleep quality did not differ between both groups during PSG; therefore, TTH may not change sleep structure. The mechanism of these findings is still unclear, and further studies should explain in detail the association between TTH and OSA.

## 1. Introduction

Tension-type headache (TTH) is one of the most common primary headaches [1,2], and its prevalence in the last three decades is still rising [3]. TTH is often a bilateral ache that can cover the entire head [1,4,5]. It is frequently described as a mild to moderate headache [4], with a lower level of intensity than other primary headaches, such as migraine [6]. The estimated occurrence of active TTH in the global population is around 26% [7], which makes it one of the most common neurological diseases [4]. TTH may be categorized as chronic or episodic, which is further divided into frequent and infrequent episodic TTH [8]. All of the mentioned types of TTH are pressing or tightening headaches with bilateral location and mild to moderate intensity. They are not aggravated by routine physical activity, and they may occur with photophobia or phonophobia but only one of them. The most significant difference between them is numbers and duration of episodes. In case of episodic TTH episodes lasting from 30 min to 7 days, in infrequent episodic TTH, there is only one episode per month, but in the case of frequent episodes, there are from two to fourteen per month. Chronic TTH evolves from episodic TTH, number of episodes rises to a minimum of 15 per month and lasts hours to days [8]. The etiology of the tension-type headache is not fully understood, but psychological disorders, genetic factors, abnormal response in the neuromodulators pathway of the central nervous system, or muscle tension are often discussed [9,10,11].

Obstructive sleep apnea (OSA) is defined as repetitive collapsing of pharyngeal soft tissues with partial or complete blockage of the airways during sleep [12,13]. OSA is manifested by snoring, fatigue, daytime sleepiness or, in more severe instances, visible and audible events of gasping or choking during sleep often witnessed by sleep partners [14]. According to the latest estimations, OSA commonly occurs in the population with a prevalence of approximately 56% worldwide [15]. OSA is a well-known factor for increased cardiovascular risk [16,17], hypertension, stroke, arrhythmias, coronary artery disease, and metabolic syndrome [16,18,19]. OSA and its comorbidities pose a serious economic burden on society [20,21].

In sleep bruxism (SB), increased activity of masseter muscles is observed, and tooth grinding may be a part of the clinical SB picture; the prevalence of SB in the general population ranges from 10 to 13 percent [22]. It is more common in younger people than in adults, and gender has no impact on its occurrence [23]. The etiology of SB is still unclear; it is postulated to be multifactorial, and different factors are considered in the development of this disorder, such as smoking, alcohol consumption, stress, and genetic predisposition [22,24]. The diagnosis of SB embraces several methods, such as subjects self-reporting symptoms using appropriate questionnaires; clinical examination of the oral cavity focusing on teeth, oral mucosa, tongue, or muscles; and finally, using PSG with electromyography (EMG) [2,25]. Nowadays, there is also a comprehensive tool that combines features such as etiology, status, consequences, and comorbidities of bruxism called STAB [25]. In clinical practice, possible bruxism is recognized only when self-reported history of SB is positive. If the dentistry evaluation reveals changes in the oral cavity, probable SB should be diagnosed. And a definite SB is when polysomnography with EMG shows masticatory muscle activity during the night [2]. Bruxism can often co-occur with other diseases. It may be linked with Parkinson’s disease, gastroesophageal reflux disease, epilepsy, and others [26,27,28,29]. There are reports of its protective mechanism in OSA, where teeth grinding is a response that increases airway patency in the course of OSA [30].

TTH, OSA, and SB are common in the global population; however, in the literature, there are only a few studies investigating these associations. In the studies by Kristiansen et al. and Chiu et al. regarding connections between OSA and TTH, the results contrasted with each other [31,32]. Chiu et al. concluded that TTH patients have an increased risk for OSA, but the likelihood was weak [31]. Additionally, a meta-analysis related to this topic shows that OSA participants did not have higher odds of TTH compared to controls [12]. A similar situation can be observed for the results of SB and TTH. Fernandes et al. observed that SB alone did not increase TTH occurrence, but SB in connection with temporo-mandibular disorder (TMD) significantly increased TTH risk [33]. A systematic review performed in 2021 confirmed results suggesting that there is no difference in TTH incidence between SB and controls [34]. However, these studies did not involve an SB diagnosis using the PSG method, and additionally, the systematic review consisted of only three articles on these issues.

Therefore, taking all these aspects into consideration, we decided to perform a new research project using diagnoses based on ICHD-3 criteria and PSG. The main objective of our study was to estimate relations between TTH and OSA or SB. Additionally, we performed an analysis of sleep structure between these groups of participants. The null hypothesis of this study is that the TTH is not related at any level to OSA or SB.

## 2. Materials and Methods

### 2.1. Eligibility Criteria

The study received approval from the Ethical Committee at Wroclaw Medical University (no. KB-25/2024, Approval date: 15 January 2024). Between 2022 and 2024, patients experiencing symptoms of SB or OSA were referred to the Sleep Laboratory at the Department and Clinic of Internal Medicine, Occupational Diseases, Hypertension, and Clinical Oncology at Wroclaw Medical University to confirm or exclude the initial diagnosis of these sleep disorders.

**Scheme 1:**
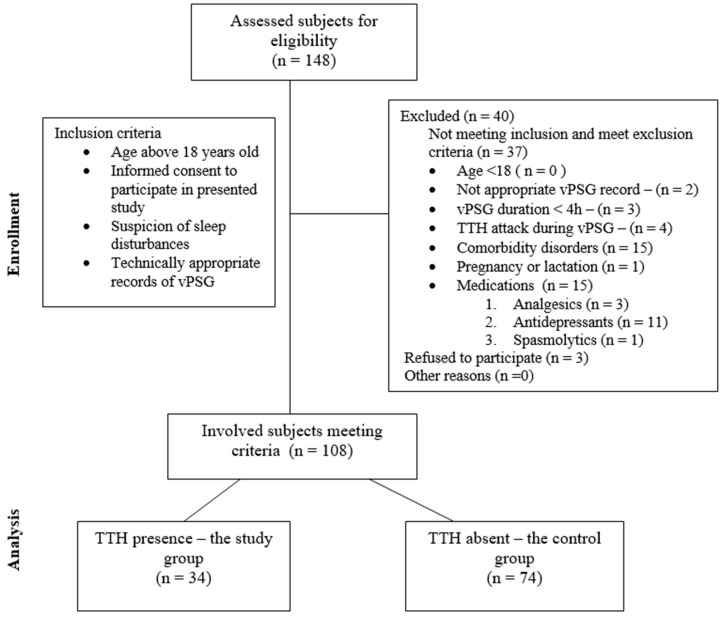
Enrollment subjects process into present study.

Inclusion criteria in the presented study included participants who had given written informed consent, were adults (above 18 years old), and who underwent single-night vPSG. Exclusion criteria were ages below 18 years, technically not appropriate nocturnal vPSG records, a total sleep time (TST) during examination below 4 h, headache attacks during the vPSG, lack of informed consent, and participants with neurological and psychiatric disorders, autoimmune and systemic diseases, malignancies, pregnancy, or lactation. Subjects using analgesics, antidepressants, and spasmolytics were excluded. The same inclusion and exclusion criteria were used for subjects from the control group. Scheme 1 presents detailed steps of subjects inclusion in this study.

### 2.2. Study Procedures

The vPSG examination was conducted using a NoxA1 device (NOX Medical, Iceland). Nocturnal recording was estimated by using the Noxturnal software (Nox Medical, Reykjavík, Iceland, version: 2.6), and it involved electroencephalographic (EEG), electrooculographic (EOG), and electrocardiographic recordings; also, abdominal and thoracic respiratory movements were recorded with body position. Oxygen saturation levels, pulse, and plethysmographic data were measured with a NONIN WristOx2 3150 pulse oximeter (Nonin Medical Inc., Plymouth, MN, USA). No teeth-protecting devices were used during the examination. The final vPSG recordings, including audio and video recording in 30-s epochs, were estimated the next morning by a physician experienced in sleep assessment (HM) according to the American Academy of Sleep Medicine (AASM) criteria for sleep scoring [35].

### 2.3. Variables

The given parameters were measured during the vPSG: BEI (bruxism episodes index)—amount of bruxism episodes per hour of sleep; ODI (oxygen desaturation index)—the average number of desaturation episodes per hour, with desaturation defined as a decrease in mean oxygen saturation of ≥3% (over the last 120 s) lasting for at least 10 s; AHI (apnea–hypopnea index)—number of apnea and hypopnea episodes per hour of sleep; TST (total sleep time)—the amount of time spent sleeping during the primary sleep period; Snore—the percentage of time spent snoring compared to TST; PLMS (periodic leg movements in sleep)—number of repetitive leg and/or arm movements per hour of sleep; SE (sleep efficiency)—the percentage of time a person spends asleep compared to the total time dedicated to sleep; WASO (wake after sleep onset)—defined as duration of time spent awake after defined sleep onset; N1, N2, N3, REM (rapid eye movement) Percentage of time spend in: stage N1 sleep, stage N2 sleep, stage N3, and rapid eye movement (REM) sleep in comparison to TST; SL (sleep latency)—the time it takes for a subject to fall asleep after the lights were turned off; REML (rapid eye movement sleep latency)—the duration between sleep onset and entering the first REM stage; Average SpO2 (%)—average O2 saturation; Minimum SpO2 (%)—lowest O2 saturation value; SpO2 duration <90% (%)—percent of sleep time with O2 saturation below <90% value.

### 2.4. Diagnostic Criteria

Apneas were defined as the lack of airflow for at least 10 s. Hypopnea was defined as a decrease in the amplitude of breathing by at least 30% for minimum 10 s with a ≥3% decline in blood oxygen saturation or arousal. In accordance with AASM [36], subjects were divided into groups due to severity: mild OSA when AHI ≥ 5 and < 15, moderate OSA when AHI ≥ 15 and <, severe OSA when AHI ≥ 30. OSA was diagnosed using the following International Classification of Sleep Disorders (ICSD-3) criteria [37]: definitely without presented symptoms if AHI ≥ 15; or AHI ≥ 5 during PSG examination with clinical symptoms such as sleepiness, nonrestorative sleep, fatigue or insomnia symptoms, or waking with breath holding, gasping, or choking during the night.

SB diagnosis was based on electromyographic (EMG) recordings from the chin area and bilaterally from the masseter muscles with audio and video recording. The bruxism episode index (BEI), phasic bruxism (more than two cyclic phasic EMG increases lasting 0.25 s–2 s), tonic bruxism (episodes lasting > 2 s), and mixed bruxism (combined both mentioned types) were used to diagnose SB. Therefore, according to AASM and Lobbezoo et al., sleep bruxism was classified as irrelevant (BEI < 2), mild to moderate (BEI 2–4), or severe (BEI > 4) based on BEI parameter [2,35].

Medical interviews conducted during the hospital stay by a qualified neurologist specializing in headache field (MWP) included the occurrence of headaches. The International Classification of Headache Disorders (ICHD-3) guidelines were used to diagnose tension-type headache [8]. Headache characteristics and treatment patterns were also obtained. Participants presenting comorbid TTH with other types of primary and secondary headache were excluded from this study.

### 2.5. Statistical Analysis

The PSG results were analyzed using Statistica v.13.3 statistical software (TIBCO Software Inc., Palo Alto, CA, USA). Quantitative variables were calculated as means (M), standard deviations (SD), medians (Me), lower quartiles (Q1), and upper quartiles (Q3), with the smallest (Min) and the largest (Max) values. The compliance of the empirical distributions of quantitative variables with the theoretical normal distribution was verified using the Shapiro–Wilk test. The significance of differences in average values (medians) of quantitative variables with non-normal distribution or with heterogeneous variances in two independent groups was verified with the Mann–Whitney U test. In the case of a larger number of groups, the Kruskal–Wallis test was used. Bartlett’s test was used to check the homogeneity of variance of the results. Qualitative variables are presented in tables as numbers (n) and proportions (%). The Pearson Chi-square test of independence was used to assess the relationships between two qualitative variables. Spearman’s rank correlation coefficient rho was used to assess the strength correlation between two quantitative variables. For all analysis, *p* < 0.05 was considered to be statistically significant.

## 3. Results

**Figure 1:**
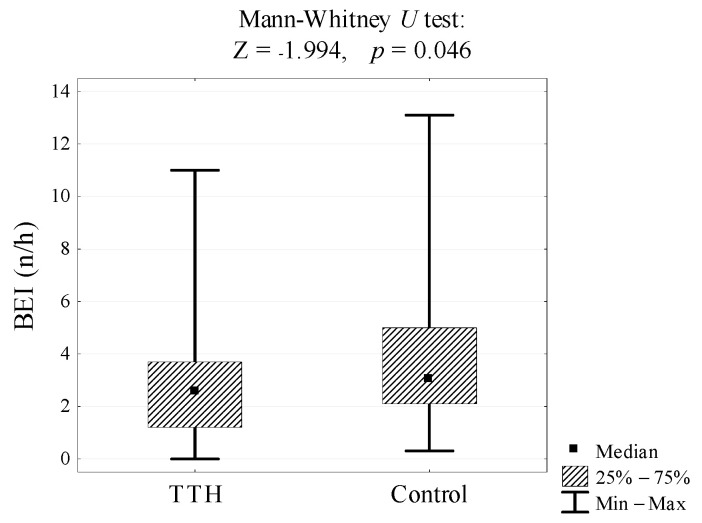
Bruxism episode index in TTH and control groups and the results of the Mann–Whitney U test.

The study involved 108 participants: 34 TTH and 74 control patients. There were 52 females and 56 males. Mean age in TTH was 40.2 ± 12.5 years old and 42.9 ± 12.9 in controls. The mean BMI in the TTH group was 38.9 ± 5.8; in the controls, it was 27.0 ± 5.1. TTH patients had had an average 11 headache days in the previous month (median = 7); therefore, all patients met criteria for episodic TTH. Each patient underwent a brain imaging examination (CT or MRI), which showed no significant abnormalities. The observed mean BEI value was 2.87 ± 2.39 in TTH subjects and 3.96 ± 2.91 in the controls. BEI value was decreased in the TTH group in comparison to the controls (2.6 n/h vs. 3.1 n/h, respectively; *p* = 0.046, Figure 1). The differences in the frequency of OSA occurrence between TTH group and control were not statistically significant (52.9% vs. 48.6%, *p* = 0.678), and no significant correlation was observed between the severity of OSA and the presence of TTH (c^2^ = 0.66, df = 3, *p* = 0.883). Table 1 presents a precise description of both groups.

None of the PSG parameters differed between TTH and control groups.

Table 2 presents a detailed description of sleep parameters in both groups.

**Figure 2:**
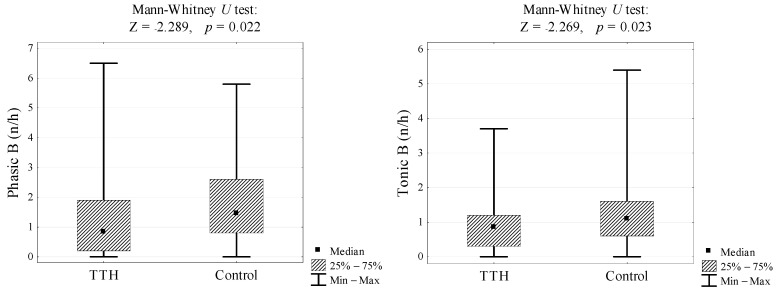
Phasic and tonic bruxism in groups differing in the occurrence of tension-type headache and the results of the Mann–Whitney U test.

In subjects with tension-type headache, phasic and tonic bruxism episodes were less frequent than in the controls (0.9 n/h vs. 1.5 n/h, *p* = 0.022 and 0.9 n/h vs. 1.1 n/h, *p* = 0.023, respectively; Table 3 and Figure 2).

There was a lack of statistical difference between severity of SB and TTH occurrence. Table 4 contains detailed information about severity of bruxism among study groups.

The study shows that tension-type headache (TTH) occurs significantly less often in people with bruxism (25.3% vs. 45.5%, *p* = 0.038, Table 5). In the TTH group, the chance of bruxism is more than two times lower compared to people without TTH (OR = 0.41), and the 95% confidence interval for OR does not include the value 1, which is interpreted as the chance of significant bruxism in both groups not being the same (Table 5). However, the chance of severe SB (BEI > 4) in TTH does not differ from that in the control group (OR = 0.54, 95% CI: 0.21–1.35; Table 6).

## 4. Discussion

Our hypothesis was partially confirmed; OSA was not connected with TTH, but TTH participants experienced SB significantly more rarely during sleep in comparison to the control patients; however, according to SB diagnosis criteria, TTH patients may qualify as having SB. However, the TTH group has less chance of SB occurrence as the control group. In the literature, there are individual studies concerning this topic. Wagner et al. [37] concluded that sleep bruxism was not a risk factor for TTH, but awake bruxism was. Fernandes et al. [33] also confirmed that SB alone was not associated with TTH. Similar to our study, the above observations were obtained for episodic TTH. However, we should emphasize that temporomandibular disorder (TMD) with concomitant SB significantly increased the risk of TTH, according to the above studies [33,37]. SB is not the same as TMD; however, SB may predispose to TMD [38]. Therefore, to exclude the potential influence of TMD on our study results, we avoided including participants with potential TMD and headaches. In addition, we avoided including TTH participants with cranial autonomic symptoms to prevent further TTH diagnosis errors [39].

However, SB affected 50% of children with TTH [40], and they had twice the risk of SB [40]. This is not a surprising conclusion because SB prevalence decreases with age [41], and in adults with TTH in our study, the prevalence of SB was 25.3%. It should be emphasized that the cited studies were, in most cases, based on questionnaires for SB diagnosis, and no PSGs were used; even the PSG study [40] did not mention methods to assess SB. The gold diagnostic standard for SB and OSA remains polysomnography [42,43]; therefore, our results could differ from those in other published studies. Additionally, many studies do not determine the precise type of headache [44]. There exists a theory explaining the association between SB and TTH based on trigger points localized around the head and neck, which are stimulated by bruxism that can generate TTH [34]. However, in the present study, the durations of SB episodes were similar between TTH and control groups, and additionally, tonic and phasic episodes were both less frequent in TTH than in the controls. Therefore, there may be a possible lack of influence of TTH on SB rather than SB exacerbating TTH. Nevertheless, we know that SB may not impact the severity of headache in a patient’s daily life [45].

The relationship between OSA and TTH has not been fully explored in the literature. In the few published papers, the results are not conclusive, because according to Chiu et al. [31], OSA individuals have a higher chance in developing TTH, and it is significantly more frequent among TTH individuals. However, this probability was 1.18, with a prevalence in 10% of participants, so the risk is barely increased [31]. Other studies demonstrated contrasting results [32]. In our paper, we discovered that the occurrence of OSA does not correlate with the presence of TTH, which is in line with Kristiansen et al.’s findings [32]. In this study the severity level of OSA also did not differ significantly between both groups, which is a similar outcome to that of Kristiansen et al. [32]. Chiu et al. [31] did not analyze discrepancies between different OSA severity groups and did not provide data about OSA severity. The inconsistencies between the findings may be caused by the differing populations included in the study. Chiu et al. [31] included patients with comorbidities such as epilepsy, major depression, and anxiety disorder, which may interfere with the results and diagnostic criteria adopted. Additionally, a recent meta-analysis about headache occurrence in OSA showed that TTH affected 19% of patients [12]. However, headaches including TTH may be relieved by OSA treatment using standard therapy, such as continuous positive airway pressure (CPAP) [46]. Further studies should be performed.

There was no difference in sleep structure measured in PSG between the TTH group and the controls, which is consistent with our hypothesis. But there are not many studies concerning sleep quality using PSG. For example, questionnaires related to sleep quality are popular for assessing sleep in TTH participants [47,48], and results have shown that TTH subjects have worse quality of sleep. However, in one of the published studies using PSG, the authors observed similar findings to those in our study, but in this study [49] TTH patients had longer slow-wave sleep (N3 stage of sleep). In addition, this study included sleep quality questionnaires; TTH subjects reported worse sleep quality and more insomnia features. Given these inconsistent observations, the authors proposed an interesting hypothesis that TTH subjects have a relative sleep deficit due to a higher need for sleep [49]. We cannot corroborate this hypothesis. It would be worth conducting further studies to extend the cited problems to include subjects’ subjective assessments of their sleep quality. For chronic TTH in Verma et al. study [50], sleep parameters in PSG showed only decreased sleep efficacy, while other parameters were normal. However, our population consisted of a majority of episodic TTH; thus, it is difficult to compare the obtained results.

Our study had some limitations. The study sample of TTH participants was insufficient and included only Caucasians; therefore, there is a potential risk of bias. Additionally, the null hypothesis was only partially explored, because a small TTH sample was included and the temporal relationship between mentioned disorders was not examined. Moreover, using the ICHD-3 criteria to diagnose TTH means there is a possibility to misdiagnose primary headaches. PSG was conducted without an adaptive night, and this should be considered when extrapolating our findings for other populations. Bruxism diagnosis was obtained by using PSG, which is still considered the main tool in the instrumentally based assessment of bruxism, but lacks other components as recommended in STAB [25].

## 5. Conclusions

Participants with diagnosed TTH had less than half the incidence of experiencing SB. Both phasic and tonic SB episodes had a lower incidence in the TTH group. The mechanism of this phenomenon is still unclear, and further research should be performed to clarify its nature. We did not observe significant differences in OSA occurrence among TTH subjects. Sleep architecture and respiratory disturbances did not differ between the TTH group and the controls. The topic of this study is still poorly understood and should be developed further.

## Figures and Tables

**Table 1:** Basic features of the study and control groups and comparison test results.

Features	TTH *N* = 34	Control *N* = 74	Results of Test
Gender:			χ^2^ = 2.93
Female	21 (61.8%)	31 (41.9%)	df = 1
Male	13 (38.2%)	43 (58.1%)	*p* = 0.087
Age (years):			
*Mean* ± *SD*	40.2 ± 12.5	42.9 ± 12.9	Z = −0.982
*Me* [*Q*1; *Q*3]	40 [31; 48]	42 [31; 55]	*p* = 0.326
*Min*–*Max*	18–66	20–65	
BMI (kg/m^2^):			
*Mean* ± *SD*	38.9 ± 5.8	27.0 ± 5.1	Z = 1.789
*Me* [*Q*1; *Q*3]	28.0 [25.4; 33.6]	26.1 [23.2; 30.5]	*p* = 0.074
*Min*–*Max*	19.5–43.7	18.4–38.1	
BEI (n/h):			
*Mean* ± *SD*	2.87 ± 2.39	3.96 ± 2.91	Z = −1.994
*Me* [*Q*1; *Q*3]	2.6 [1.2; 3.7]	3.1 [2.1; 5.0]	*p* = 0.046
*Min*–*Max*	0.0–11.0	0.3–13.1	
Bruxism episodes index:			
BEI < 2	14 41.2%	17 32.0%	χ^2^ = 4.06
2 ≤ BEI ≤ 4	12 35.3%	30 40.5%	df = 2
BEI > 4 BEI > 2	8 23.5% 20 58.8%	27 36.5% 57 77.0%	*p* = 0.131 *p* = 0.068
Obstructive sleep apnea			
AHI < 5	16 47.1%	38 48.6%	*c^2^* = 0.66
AHI 5–15	9 26.4%	17 23.0%	*df* = 3
AHI 15–30	5 14.7%	8 10.8%	*p* = 0.883
AHI > 30	4 11.8%	11 14.9%	

Abbreviations: SD standard deviation; Me—median (50%); Q1 lower quartile (25%); Q3 upper quartile (75%); Min smallest value; Max highest value; *p* significance level of the statistical test, Z, Mann-Whitney U test statistic, BEI—bruxism episode index.

**Table 2:** PSG parameters in TTH and control groups.

PSG Parameters	TTH *N* = 34	Control *N* = 74	Results of Test
AHI (n/h):			
*Mean* ± *SD*	12.3 ± 17.5	12.2 ± 16.7	Z = −0.212
*Me* [*Q*1; *Q*3]	5.7 [1.9; 19.0]	4.7 [2.5; 14.0]	*p* = 0.832
*Min*–*Max*	0.3–85.8	0.4–77.7	
ODI (n/h):			
*Mean* ± *SD*	13.1 ± 18.2	11.9 ± 15.5	Z = 0.026
*Me* [*Q*1; *Q*3]	7.3 [2.4; 20.6]	5.9 [3.0; 12.6]	*p* = 0.979
*Min*–*Max*	0.4–94.8	0.6–71.5	
Snore (% of TST):			
*Mean* ± *SD*	16.8 ± 19.5	14.4 ± 17.2	Z = 0.410
*Me* [*Q*1; *Q*3]	11.5 [0.3; 27.4]	7.6 [0.5; 21.7]	*p* = 0.682
*Min*–*Max*	0.0–78.1	0.0–65.3	
PLMS (n/h):			
*Mean* ± *SD*	9.1 ± 19.5	4.2 ± 6.8	Z = 1.634
*Me* [*Q*1; *Q*3]	3.6 [0.1; 9.2]	1.7 [0.0; 5.9]	*p* = 0.102
*Min*–*Max*	0.0–111.6	0.0–31.7	
TST (min):			
*Mean* ± *SD*	426 ± 68	439 ± 59	Z = −1.039
*Me* [*Q*1; *Q*3]	435 [397; 490]	456 [418; 471]	*p* = 0.299
*Min*–*Max*	276–545	211–535	
SL (min):			
*Mean* ± *SD*	15.1 ± 13.0	15.9 ± 14.4	Z = 0.142
*Me* [*Q*1; *Q*3]	11.6 [7.1; 17.9]	12.1 [5.6; 21.6]	*p* = 0.887
*Min*–*Max*	0.4–63.8	0.3–68.5	
REML (min):			
*Mean* ± *SD*	83.6 ± 40.3	81.6 ± 44.7	Z = 0.648
*Me* [*Q*1; *Q*3]	71.5 [64.0; 95.0]	71.1 [52.5; 97.0]	*p* = 0.517
*Min*–*Max*	0.0–202.1	0.0–265.0	
WASO (min):			
*Mean* ± *SD*	43.3 ± 28.6	41.6 ± 34.9	Z = 0.384
*Me* [*Q*1; *Q*3]	34.7 [22.8; 66.6]	35.5 [22.8; 50.0]	*p* = 0.701
*Min*–*Max*	3.0–118.5	0.5–233.5	
SE (%):			
*Mean* ± *SD*	87.5 ± 8.2	88.1 ± 7.8	Z = −0.212
*Me* [*Q*1; *Q*3]	91.0 [80.7; 93.0]	90.5 [85.4; 93.0]	*p* = 0.832
*Min*–*Max*	67.9–97.8	50.4–97.9	
N1 (% of TST):			
*Mean* ± *SD*	5.4 ± 4.6	5.4 ± 4.7	Z = 0.040
*Me* [*Q*1; *Q*3]	3.9 [2.3; 6.2]	3.8 [2.1; 6.6]	*p* = 0.968
*Min*–*Max*	0.8–16.8	0.2–20.7	
N2 (% of TST):			
*Mean* ± *SD*	47.4 ± 9.9	51.0 ± 8.5	Z = −1.574
*Me* [*Q*1; *Q*3]	48.0 [42.9; 53.7]	52.1 [44.7; 56.0]	*p* = 0.115
*Min*–*Max*	22.0–66.1	30.3–76.0	
N3 (% of TST):			
*Mean* ± *SD*	25.1 ± 12.8	21.6 ± 9.2	Z = 1.134
*Me* [*Q*1; *Q*3]	21.6 [19.0; 26.7]	20.6 [16.6; 25.7]	*p* = 0.257
*Min*–*Max*	13.9–85.5	0.0–64.6	
REM (% of TST):			
*Mean* ± *SD*	23.5 ± 6.0	22.0 ± 6.1	Z = 0.953
*Me* [*Q*1; *Q*3]	23.7 [19.0; 28.7]	22.7 [19.4; 26.0]	*p* = 0.341
*Min*–*Max*	11.5–36.2	0.0–35.4	
Average SpO_2_ (%):			
*Mean* ± *SD*	93.5 ± 2.1	93.1 ± 5.3	Z =- 0.162
*Me* [*Q*1; *Q*3]	93.8 [92.5; 94.9]	94.4 [91.8; 94.9]	*p* = 0.871
*Min*–*Max*	86.9–96.3	51.0–96.7	
Minimum SpO_2_ (%):			
*Mean* ± *SD*	84.3 ± 8.6	83.8 ± 6.5	Z = 1.340
*Me* [*Q*1; *Q*3]	87.5 [82.0; 89.0]	84.0 [80.0; 88.0]	*p* = 0.180
*Min*–*Max*	56.0–94.0	58.0–95.0	
SpO_2_ duration <90% (%):			
*Mean* ± *SD*	7.3 ± 16.8	6.3 ± 11.1	Z = −0.648
*Me* [*Q*1; *Q*3]	0.5 [0.0; 5.9]	0.7 [0.1; 8.5]	*p* = 0.517
*Min*–*Max*	0.0–72.4	0.0–52.9	

Abbreviations: TST, total sleep time; SE, sleep efficiency; REML, rapid eye movement sleep latency; WASO, wake after sleep onset; N1, N2, N3; REM, rapid eye movement; PLMS, Periodic leg movements in sleep; SL, sleep latency; SpO2, saturation O2; ODI, oxygen desaturation index; AHI, apnea–hypopnea index; M, mean; SD, standard deviation; Me, median (50%); Q1, lower quartile (25%); Q3, upper quartile (75%); Min, minimum value; Max, maximum value; Z, Mann-Whitney U test statistic; *p*, significance level of the statistical test.

**Table 3:** Detailed bruxism parameters obtained during PSG in TTH and control groups.

Bruxism Parameters	TTH *N* = 34	Control *N* = 74	Results of Test
Phasic B (n/h):			
*Mean* ± *SD*	1.37 ± 1.65	1.85 ± 1.49	Z = −2.289
*Me* [*Q*1; *Q*3]	0.9 [0.2; 1.9]	1.5 [0.8; 2.6]	*p* = 0.022
*Min*–*Max*	0.0–6.5	0.0–5.8	
Tonic B (n/h):			
*Mean* ± *SD*	0.88 ± 0.77	1.44 ± 1.32	Z = −2.269
*Me* [*Q*1; *Q*3]	0.9 [0.3; 1.2]	1.1 [0.6; 1.6]	*p* = 0.023
*Min*–*Max*	0.0–3.7	0.0–5.4	
Mixed B (n/h):			
*Mean* ± *SD*	0.48 ± 0.39	0.66 ± 0.68	Z = −0.900
*Me* [*Q*1; *Q*3]	0.4 [0.2; 0.7]	0.5 [0.2; 0.9]	*p* = 0.368
*Min*–*Max*	0.0–1.4	0.0–3.5	
BEI average (s)			
*Mean* ± *SD*	6.63 ± 3.16	6.89 ± 2.12	Z = −0.806
*Me* [*Q*1; *Q*3]	6.2 [4.4; 8.4]	6.7 [5.2; 8.1]	*p* = 0.420
*Min*–*Max*	0.0–15.7	3.3–11.8	
Phasic average (s)			
*Mean* ± *SD*	6.48 ± 3.84	7.77 ± 3.52	Z = −1.549
*Me* [*Q*1; *Q*3]	6.1 [4.2; 9.9]	7.5 [5.5; 10.1]	*p* = 0.121
*Min*–*Max*	0.0–14.5	0.0–17.1	
Tonic average (s)			
*Mean* ± *SD*	3.41 ± 1.21	3.96 ± 2.91	Z = −1.489
*Me* [*Q*1; *Q*3]	3.3 [2.8; 3.7]	3.5 [3.0; 4.3]	*p* = 0.137
*Min*–*Max*	0.0–6.3	0.0–8.6	
Mixed average (s)			
*Mean* ± *SD*	6.95 ± 4.89	8.02 ± 5.03	Z = −1.047
*Me* [*Q*1; *Q*3]	6.7 [3.9; 10.2]	8.5 [5.6; 10.7]	*p* = 0.297
*Min*–*Max*	0.0–17.4	0.0–29.7	

Abbreviations: M mean; SD standard deviation; Me—median (50%); Q1 lower quartile (25%); Q3 upper quartile (75%); Min smallest value; Max highest value; *p* significance level of the statistical test, Z, Mann-Whitney U test statistic, BEI—bruxism episode index.

**Table 4:** Number (percentage) of participants in groups differing in the severity of bruxism and the occurrence of tension headache and comparative results (Pearson’s chi-square test).

Group	Irrevelevant BEI < 2 *N* = 31	Mild/Moderate 2 ≤ BEI ≤ 4 *N* = 42	Severe BEI > 4 *N* = 35	Test Results
TTH				χ^2^ = 4.06
Yes, *n* (%)	14 (45.2)	12 (28.6)	8 (22.9)	df = 2
No, *n* (%)	17 (54.8)	30 (71.4)	27 (77.1)	*p* = 0.131

Abbreviations: TTH, tension-type headache; *p* significance level of the statistical test, BEI—bruxism episode index.

**Table 5:** Number (percentage) of people in groups differing in the occurrence of significant bruxism and tension-type headache, as well as the independence test result and odds ratio with 95% confidence interval.

Group	BEI < 2 *N* = 33	BEI > 2 *N* = 75	*p*-Value	OR (95% CI)
TTH	19 (25.3%)	15 (45.5%)	0.038	0.41 (0.17–0.96)
Control	56 (74.7%)	18 (54.5%)		1.00 (ref.)

Abbreviations: TTH, tension-type headache; *p* significance level of the statistical test, BEI—bruxism episode index; OR, odds ratio.

**Table 6:** Number (percentage) of people in groups differing in the type of bruxism and the occurrence of tension headache and comparative results (Pearson’s chi-square test).

Group	BEI ≤ 4 *N* = 73	BEI > 4 *N* = 35	*p*-Value	OR (95% CI)
TTH	26 (35.6%)	8 (22.9%)	0.181	0.54 (0.21–1.35)
Control	47 (64.4%)	27 (77.1%)		1.00 (ref.)

Abbreviations: TTH, tension-type headache; *p* significance level of the statistical test, BEI—bruxism episode index; OR, odds ratio.

## Data Availability

The dataset is available upon request from the authors.
